# Robust and automatic beamstop shadow outlier rejection: combining crystallographic statistics with modern clustering under a semi-supervised learning strategy

**DOI:** 10.1107/S2059798324008519

**Published:** 2024-10-01

**Authors:** Yunyun Gao, Helen M. Ginn, Andrea Thorn

**Affiliations:** a Insitut für Nanostruktur und Festkörperphysik Universität Hamburg Hamburg Germany; b Center for Free-Electron Laser Science, CFEL Deutsches Elektronen-Synchrotron (DESY) Germany; University of Cambridge, United Kingdom

**Keywords:** X-ray crystallography, *AUSPEX*, outliers, statistics, clustering, semi-supervised learning

## Abstract

During the automatic processing of crystallographic diffraction experiments, beamstop shadows are often unaccounted for or only partially masked. As a result of this, outlier reflection intensities are integrated, which is a known issue. Traditional statistical diagnostics have only limited effectiveness in identifying these outliers, here termed Not-Excluded-unMasked-Outliers (NEMOs). The diagnostic tool *AUSPEX* allows visual inspection of NEMOs, where they form a typical pattern: clusters at the low-resolution end of the *AUSPEX* plots of intensities or amplitudes versus resolution. To automate NEMO detection, a new algorithm was developed by combining data statistics with a density-based clustering method. This approach demonstrates a promising performance in detecting NEMOs in merged data sets without disrupting existing data-reduction pipelines. Re-refinement results indicate that excluding the identified NEMOs can effectively enhance the quality of subsequent structure-determination steps. This method offers a prospective automated means to assess the efficacy of a beamstop mask, as well as highlighting the potential of modern pattern-recognition techniques for automating outlier exclusion during data processing, facilitating future adaptation to evolving experimental strategies.

## Abstract 2

Beamstops and beamstop shadows can lead to erroneous reflection measurements in crystallographic diffraction experiments, which is a common problem in data processing. Here, a method is presented that combines crystallographic statistics with machine learning to robustly detect such reflections.

## 1. Introduction

In macromolecular X-ray crystallography, determining the high-resolution cutoff for data processing is a decisive aspect of structure determination. As a result, tools have been developed to assist in identifying the optimal high-resolution cutoff for any given data set (Diederichs & Karplus, 2013 ▸; Karplus & Diederichs, 2012 ▸). However, the issue of selecting an appropriate low-resolution cutoff remains largely unaddressed as a consequence of an unfortunately common belief that low-angle data do not contribute significant details to the final model. It is true that low-angle observations in reciprocal space primarily contribute to identifying the macromolecule/solvent boundary in real space and, assuming that perfect phases have been obtained, low-angle data may indeed appear to be less pertinent to the quality of the final macromolecular model (Diederichs, 2010 ▸). Yet, achieving a (near) perfect estimate of phases is a nontrivial task. In practice, these low-angle or very low-angle observations play an important role in indexing, phasing and model refinement (Wlodawer *et al.*, 2008 ▸). Moreover, low-angle observations are crucial to indexing efficiency for serial crystallography (Li *et al.*, 2019 ▸; Nam, 2022 ▸), reciprocal-space solvent flattening (Terwilliger, 1999 ▸), bulk-solvent scaling (Afonine *et al.*, 2013 ▸), novel iterative *ab initio* phasing (Jiang *et al.*, 2023 ▸; Yoshimura *et al.*, 2016 ▸) and the biological interpretation of the partially ordered solvent interface (Dauter & Wilson, 2012 ▸; Lang *et al.*, 2014 ▸). Thus, while low-angle data should not be arbitrarily discarded, neither can the influence of systematic errors as a result of imperfect intensity estimation due to the beamstop in the low-angle region of the diffraction pattern be ignored.

**Figure 1:**
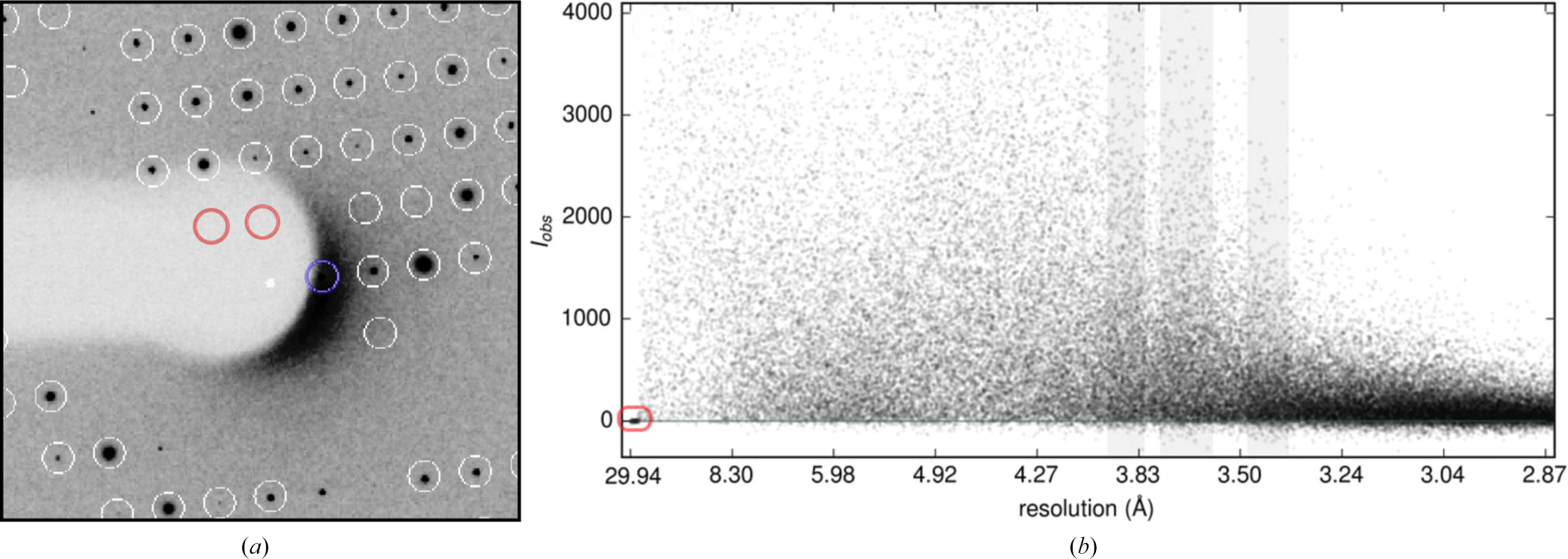
(*a*) Two types of low-angle outliers related to the beamstop. Predicted locations of reflections are circled. The blue circle and red circles highlight the type 1 and type 2 outliers, respectively. (*b*) *AUSPEX* plot of *I*_obs_ versus resolution. Unexcluded type 2 beamstop shadow outliers with intensity values near 0 cluster at the low-resolution end, indicating that the beamstop was either not masked or not masked completely prior to integration.

At low angle (>10 Å), two types of outliers (Evans, 2006 ▸) related to the beamstop emerge (Fig. 1 ▸*a*): (1) observations overlapping with the noncrystallographic scattering around the central beamstop and (2) those within the area of the diffraction pattern shadowed by the beamstop. The former are typically strong and detectable using Wilson statistics. As a result, the rejection of rogue data stemming from outliers of type 1 has been integrated into data-reduction programs, including *AIMLESS* (Evans & Murshudov, 2013 ▸), *DIALS* (Winter *et al.*, 2018 ▸) and *XDS* (Kabsch, 2010 ▸). On the other hand, the detection and exclusion of outliers of type 2 remains more difficult, although the problem has long been recognized (Read, 1999 ▸). When examining World Wide Protein Data Bank (wwPDB) entries with available raw images, we consistently observed clusters of weak signals in the lowest resolution bins in *AUSPEX* plots of intensities (*I*_obs_) and structure factors (*F*_obs_) (Fig. 1 ▸*b*). More detailed analysis of the raw images of the corresponding entries suggests that the physically obstructed areas of the beamstops are often off-centre or possess non-circular geometries. Although it is hard to reconstruct whether the depositors explicitly modelled the beamstop mask during data reduction, re-integration of these data sets reveals that these clustered low-angle weak signals indeed originate from type 2 outliers. Importantly, type 2 outliers appear not to have been rejected by the original depositors, suggesting the structure determination and model building/refinement might have been attempted with this pathology present in the data. Here, we analyse the source of low-angle outliers, termed Not-Excluded-unMasked-Outliers (NEMOs), propose a novel method for their automatic identification and assess their impact on refinement.

### 1.1. NEMOs: the outliers escaping outlier rejection

**Figure 2:**
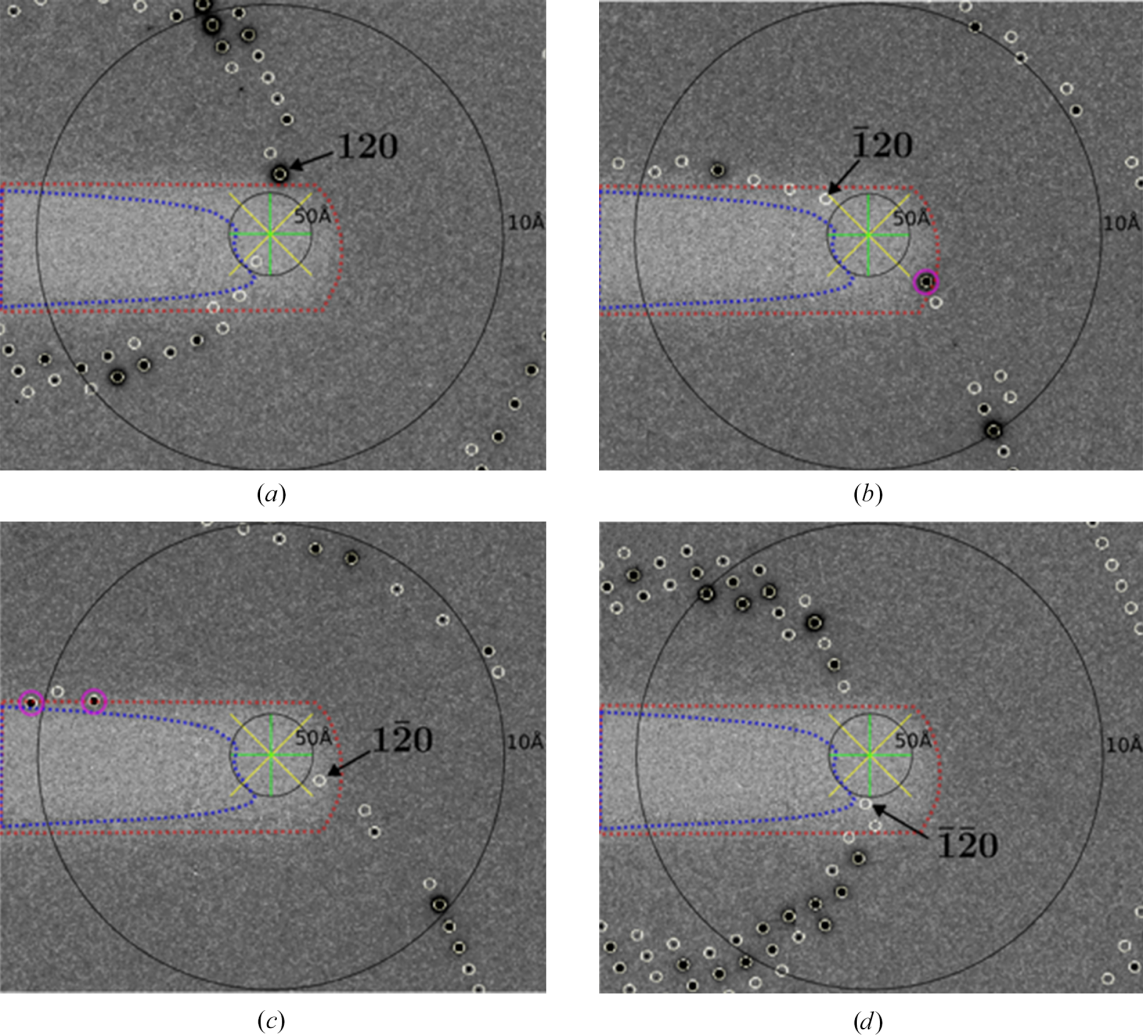
Example of the experimental origin of NEMOs. The images are the corresponding raw detector frames of PDB entry 5b8f. The blue dashed polygon highlights the edge of excluded shaded regions recognized by the best possible *XDS*–*DEFPIX* trial without masking unobstructed areas on the detector. The red dashed polygon highlights the edge of how one would intuitively define a mask, manually created with the aid of *dials.image_viewer*. The beam centre is indicated by the yellow and green crosses. The 50 and 10 Å resolution shells are marked with black rings. Predicted locations of reflections are circled in yellow. Using the blue mask, the similarity of observations 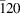 in (*b*), 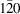 in (*c*) and 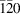 in (*d*) result in the exclusion of observation 120 in (*a*). The resulting merged unique reflection 120 would then have a value close to 0, but an ‘algorithm-acceptable’ uncertainty. Using the red mask, the unique reflection 120 can be properly recorded, as the other symmetry-equivalent observations are completely masked. However, in (*b*) and (*c*) such a mask results in the masking of unbiased observations (highlighted with magenta circles).

The conventional approach to identify an outlier intensity is to collect data of high multiplicity. In cases of multiple measurement of symmetry-related observations, the fundamental assumption is that the majority of these observations are reliable. However, this assumption may fail for low-angle data obstructed by beamstop shadows (Evans, 2011 ▸). Fig. 2 ▸ shows an example in which, of four symmetry-equivalent observations, three are behind the unmasked portion of the beamstop. Such a set of symmetry-equivalent observations leads to the exclusion of the sole correctly recorded reflection during scaling. Consequently, the merged data point becomes the average of the three improperly estimated reflection intensities.

Upon merging, detection of NEMOs is possible using CC_1/2_, as both off-centred and noncircular beamstops can introduce non-isomorphism into observations and CC_1/2_ detects this. However, this approach may fail in certain cases where NEMOs and strong observations coexist within the same selected resolution shell (Supplementary Fig. S1). In this case, the variance of the data is much larger than the variance of the multiple observations, resulting in a CC_1/2_ close to 1 in the low-resolution shell. Similar challenges can be encountered with other statistical tools where binning is imperative.

The persistence of NEMOs as clusters of weak signals is exacerbated as a result of applying the French–Wilson method when estimating the structure factors from the intensities (French & Wilson, 1978 ▸). Given the high likelihood of weak intensities in the Wilson distribution, the posterior distribution for weak reflections with significant errors, such as NEMOs, tends to be dominated by the prior Wilson distribution (Read, 1999 ▸; Read & McCoy, 2016 ▸). For amplitudes that are inferred from the Wilson distribution, the minimum ratios of the French–Wilson posterior amplitude and standard deviation for centric and acentric data are 1.324 and 1.913, respectively (Read & McCoy, 2016 ▸). Upon inspecting Protein Data Bank (PDB) entries, all NEMO clusters slightly surpass these lower limits.

NEMOs can be marked as outliers at the end of each structure model-refinement cycle using model-based χ^2^ distributions (Read, 1999 ▸). *phenix.refine* (Adams *et al.*, 2010 ▸) has implemented this functionality. However, despite being flagged, the effects may persist in subsequent refinement stages, potentially undermining the accurate understanding of biological activity at the partially ordered solvent interface (Yu *et al.*, 1999 ▸; Dauter & Wilson, 2012 ▸).

The *cctbx* package offers a probability test to reject weak observations below 10 Å based on Wilson statistics, employing a loose threshold (*i.e.* 10^−2^) as opposed to the recommended criteria of 10^−6^ (Read, 1999 ▸). This empirical criterion may pose issues in detection accuracy and specificity, leading to an unwanted loss of information related to the low-angle data.

### 1.2. *AUSPEX* plots can be used to identify NEMOs

Traditionally, the assessment of diffraction data quality relies on statistical indicators, which are effective in evaluating either the overall data quality or the data quality within specific resolution bins, presuming that most data conform to an expected behaviour. Based on established conventional *ad hoc* criteria, particular subsets of the data set are then discarded as they are deemed to be non-informative or highly uncertain. However, the presence of outliers can significantly skew the behaviour of these quality indicators (Dalton *et al.*, 2022 ▸). This issue is particularly pronounced for low-angle data, where reflections tend to be sparse.

*AUSPEX* is a graphical diagnostic tool for identifying diffraction data pathologies and is commonly used, for example, to automatically detect ice rings (Thorn *et al.*, 2017 ▸; Nolte *et al.*, 2022 ▸). It exploits the fact that systematic errors consistently bias observations, thereby manifesting explicit patterns across the data set (Gao *et al.*, 2023 ▸). In an integrated, scaled and merged data set, inadequately masked beamstops often result in clusters of weak observations at the low-resolution end of the data sets. While diagnostic tools based on binning may lack the sensitivity to detect these clusters, it is intuitive for human observers to identify NEMOs as clusters within the inherently sparse low-resolution region in an *AUSPEX* plot. Two key questions arise. (i) Can such clusters be identified automatically? (ii) Are the elements in the automatically identified clusters NEMOs and only NEMOs? To address these questions, we explored a route that combines X-ray crystallographic statistics with modern density-based clustering methods and utilized a semi-supervised training strategy to further improve the robustness.

## 2. Methods

### 2.1. Automatic detection of NEMOs

A low-angle (*d*-spacing > 10 Å) data subset, ***A***, is created consisting of 2D coordinates (*x*, *y*), where *x* is the inverse *d*-spacing squared and *y* is the merged signal-to-noise ratio *F*_obs_/σ(*F*_obs_) or *I*_obs_/σ(*I*_obs_). The *d*-spacing squared is a natural choice for uniformity given that the volume of a thin shell of a sphere can be approximated quadratically (Singer, 2021 ▸). The rationale behind using merged *F*_obs_/σ(*F*_obs_) and *I*_obs_/σ(*I*_obs_) is that, unlike reflections affected by other systematic errors (Assmann *et al.*, 2016 ▸), the signal-to-noise ratio of beamstop shadow outliers does not increase after merging. In the case of intensities, *I*_obs_/σ(*I*_obs_) exhibits non-monotonic growth due to the presence of negative values resulting from the integration of predicted reflections under the beamstop shadow. When considering amplitudes, applying the French–Wilson method results in the inferred *F*_obs_ and σ(*F*_obs_) of beamstop shadow outliers predominantly being influenced by the prior distribution, which effectively amplifies the posterior error estimates σ(*F*_obs_). Therefore, the use of a merged signal-to-noise ratio is preferable. Hence,
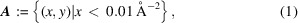


#### 2.1.1. Identifying an initial pool of potential outliers

The statistical tests proposed by Read (Read, 1999 ▸; Read & McCoy, 2016 ▸) are used to select the potential outliers. The cumulative probability functions in the form of amplitudes and intensities, respectively, are defined as follows.

For acentric reflections:
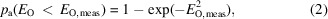

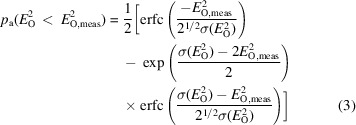


For centric reflections:
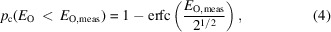

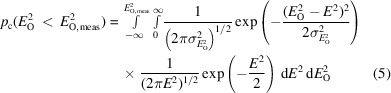


In these equations, erfc is the complementary error function, *E*_O_ is the normalized amplitude, 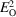 is the normalized intensity and 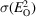 is the standard deviation for the normalized intensity. The functions compute the probability that an observation could be smaller than *E*_O,meas_ or 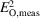. In our study, the normalization to an absolute scale is performed with the kernel normalization method in the *cctbx* library. The integration for centric intensities 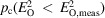 is determined numerically. The numerical integration is conducted using *nquad* in the *SciPy* library (Virtanen *et al.*, 2020 ▸) via low-level callable C functions.

For all the (*x*, *y*) in ***A***, those with cumulative probabilities smaller than *t* are marked and compose the initial potential outliers set ***O*** with a size of *k*,
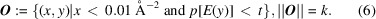


The parameter *t* is a factor deciding the tolerance to potential outliers. Set ***O*** may be a close approximation to the true set of beamstop shadow outliers when a case-specific *t* is carefully selected. Automatic detection is hard to achieve if a universal *t* threshold is applied. For example, the outlier-detection module in *cctbx* empirically classifies observations with *t* < 10^−2^ as beamstop shadow outliers, which gives unpromising results (discussed in Section 3.1).

#### 2.1.2. Clustering estimations at multiple noise levels

The hierarchical density-based spatial clustering of applications with noise (*HDBSCAN*) algorithm is a clustering algorithm that is particularly effective in identifying clusters within data sets characterized by varying densities and non-flat geometry (Campello *et al.*, 2013 ▸, 2015 ▸). Clusters observed at the low-resolution end of *AUSPEX* plots typically exhibit non-flat geometries, *i.e.* local densities significantly surpassing the neighbouring background and overall non-convex shapes. In *HDBSCAN*, the key parameter governing its operation is the *minimum cluster size* (ρ_mcs_), which serves as an abstract proxy for noise levels under examination. If a core cluster is present, the elements identified within it or its hierarchical subclusters by *HDBSCAN* exhibit mutual consistency across a broad range of minimum cluster sizes (McInnes & Healy, 2017 ▸; Guo *et al.*, 2021 ▸). Consider that set ***A*** contains a core cluster comprising beamstop shadow outliers (possibly null, representing zero beamstop shadow outliers) and noise background formed by normal data with unknown noise levels. Using a fixed, universal threshold for ρ_mcs_ may lead to either over-segmentation or under-segmentation. To address this, a series of clustering-estimation cycles are conducted, varying the ρ_mcs_ from 1 to *k*, resembling a bootstrapping approach to resample the noise level. For cluster *i* in a given clustering estimation cycle *j* (***C**_i,j_*), the population of the intersection ratio between ***C**_i,j_* and ***O*** is then calculated as
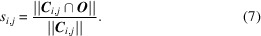


*s_i,j_* serves as a simple measurement of set overlap between ***C**_i,j_* and ***O***.

Without loss of generality, *x* is scaled to match the range of *y* to aid the use of Euclidean distance to support clustering, which is calculated as
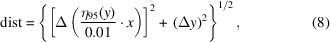
where η_95_(*y*) is the 95th percentile of *y*.

#### 2.1.3. Robust assignment of beamstop shadow outliers

To improve the robustness of assignment, the following steps are formulated based on mutual regulation. (i) An element (*x*, *y*) in set ***O*** is not classified as a NEMO if it does not show persistent membership of multiple estimated ***C**_i,j_* across different noise levels. (ii) An element (*x*, *y*) disjoint from ***O*** is classified as a NEMO if it is part of multiple estimated ***C**_i,j_* that exhibit a sufficient overlapping ratio to ***O*** across different noise levels.

Let the collective set 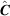 be the concatenation of ***C**_i,j_* under the condition that the elements of ***C**_i,j_* that are encompassed in ***O*** populate ***C**_i,j_* with a proper fraction of *l*,
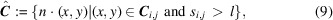
where *n* denotes the number of times that an observation is assigned as a component of a cluster during successive estimation cycles. The parameter *l* regulates the required similarity for ***C**_i,j_* to be considered as a subgroup of the core cluster formed by beamstop shadow outliers.

Finally, an observation is categorized as NEMO if *n*/*n*_max_ ≥ *m*, where *n*_max_ is the maximum multiplicity of (*x*, *y*) in 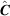 and *m* is a *d*-spacing-dependent 3-tuple determining the difficulty of satisfying the aforementioned principles. The set of NEMOs can then be represented as
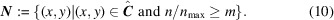


### 2.2. Hyperparameter optimization

Clustering or a clustering task-involving framework is typically viewed as an unsupervised task. However, with known ground truth and appropriate external metrics, it is possible to transition the problem into a semi-supervised regime, facilitating the tuning of hyperparameters to optimize performance. In the algorithm discussed above, the parameters *t*, *l* and *m* can be considered as hyperparameters with unit intervals that affect the performance of detection. A total of 109 Protein Data Bank (PDB) entries with available raw diffraction images were selected for hyperparameter optimization (Supplementary Information S1.1 and S1.2, list of data sets and assignment). All entries were examined using *phenix.xtriage* (Adams *et al.*, 2010 ▸) to confirm the absence of twinning or translational noncrystallographic symmetry (tNCS). To find the exact source of the weak observations, we performed re-integration using the respective raw diffraction images. The re-integration was conducted using *XDS* (version Jun 30, 2023). The space group was kept the same as that in the deposited data header and the unit-cell parameters were rounded to the nearest tenths of the reported values. The default value of 50 Å was used as the low-resolution limit. The lower bound of VALUE_RANGE_FOR_TRUSTED_DETECTOR_PIXELS was adjusted to 2000 to ensure that all predicted reflections were integrated. A validation set of NEMO labels was made manually according to the following protocol: low-angle observations below the 10 Å threshold were considered to be NEMO candidates if they (i) exhibited a poor correlation factor (<20) between the observed and the expected reflection integration profiles in the re-integrated unmerged data (column 11 in INTEGRATE.HKL) and (ii) were verified as NEMOs upon manual visual inspection of the corresponding detector frame. This inspection was carried out to check that the candidate observations were indeed predicted to be under the beamstop, which is visible using *XDS-Viewer* (Brehm *et al.*, 2023 ▸). The resulting NEMO-labelled observations from the 109 PDB entries comprise a ground-truth set. Clustering was performed using the *HDBSCAN* module within the *scikit-learn* package (Pedregosa *et al.*, 2011 ▸), with Euclidean distance between individual points (*x*, *y*) serving as the distance metric. The search and evaluation are implemented in a similar way as described in a previous study (Mishra *et al.*, 2022 ▸). The detailed tuning process is reported in Supplementary Fig. S2.

### 2.3. Performance test

**Figure 3:**
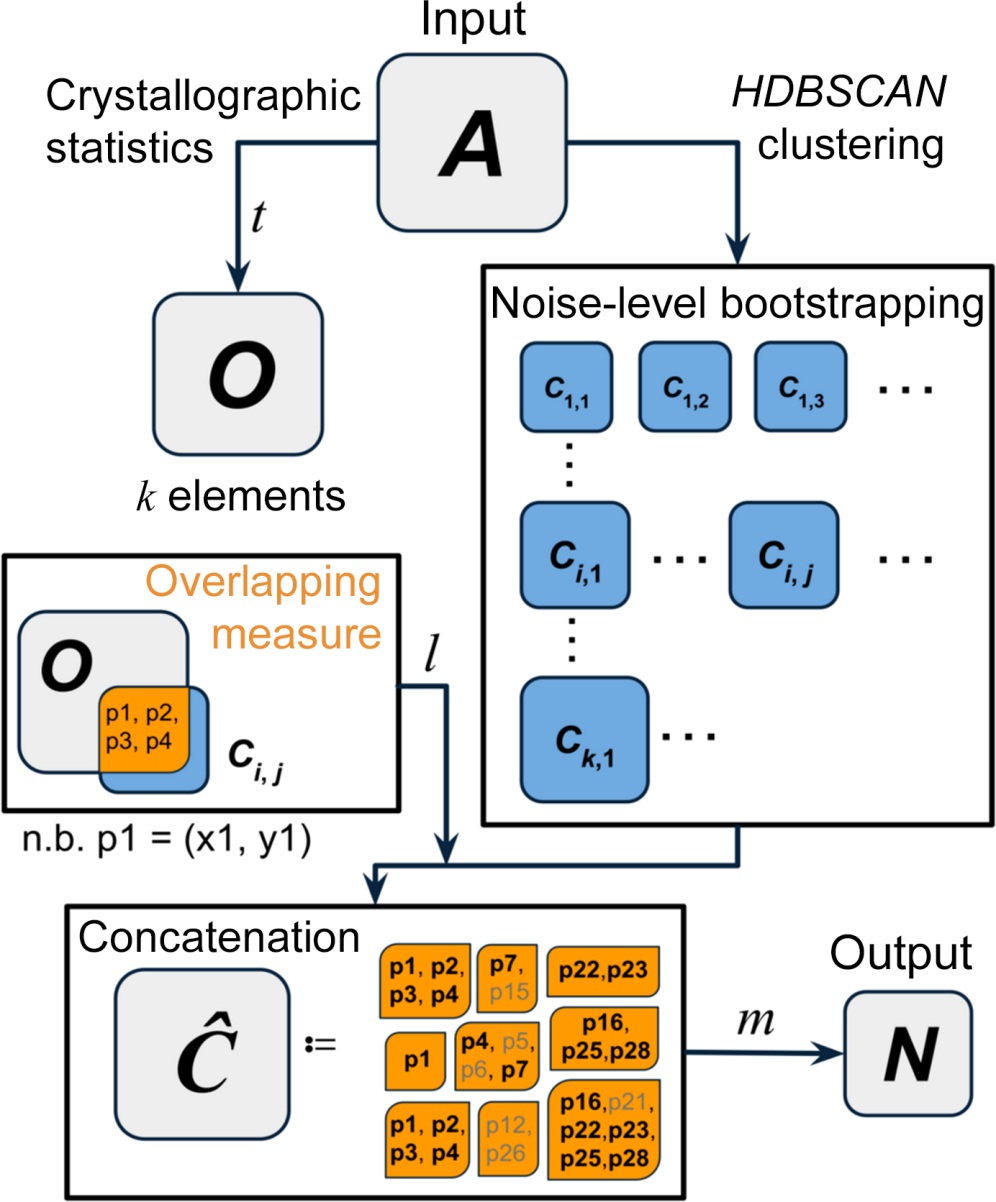
Schematic workflow of the automatic detection algorithm. The process begins with a low-angle data subset ***A***. Subsequently, crystallographic statistics and clustering bootstrapping are performed independently. The overlap between each subcluster ***C**_i,j_* and the set ***O*** derived from crystallographic statistics is then assessed. This results in the concatenated multiset 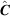, where elements can be recurrent with a certain multiplicity. If an unique element has sufficient multiplicity, it will be included in the output set ***N*** and categorized as a NEMO. The detection performance is influenced by the hyperparameters *t*, *l* and *m*. Here, an element *p* is equivalent to indexed 2D coordinates with (*x*, *y*) as positional properties in a Euclidean plane.

To evaluate the reliability of the hyperparameter-tuned automatic detection (Fig. 3 ▸), a performance test was performed with deposited data. The deposited merged data from 328 further PDB entries, each with publicly available raw diffraction images, were used to validate and assess the performance of automatic detection methods (Supplementary Information S1.1 and S1.2, list of data sets and assignment). Among these data sets, 45 exhibit the presence of twinning or/and tNCS. The same approach as outlined in Section 2.2 was employed to populate the ground-truth set ***G***.

For a given NEMO assignment ***N***, the true-positive (TP), false-positive (FP), true-negative (TN) and false-negative (FN) classifications are defined as described in Table 1 ▸.

The performance of the algorithms was evaluated using four metrics, precision, accuracy, sensitivity and specificity, which are defined as follows:
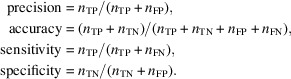


Here, *n* represents the total number of corresponding classifications across all test data sets. Accuracy measures the true concentration of beamstop shadow outliers in a given NEMO assignment. Precision measures algorithm stability towards various data sets. Sensitivity and specificity measure the abilities to correctly identify beamstop shadow outliers and to correctly exclude weak observations that are not beamstop outliers, respectively. It is worth noting that TN can be artificially inflated if more observations exist in set ***A*** (for example a larger unit cell or higher symmetry). To mitigate this effect, ***O***′ is introduced to include only nominally weak signals when counting TN.

### 2.4. Re-refinement

*PDB-REDO* (version 8.04; Joosten *et al.*, 2012 ▸) was used to benchmark the influence of NEMOs on refinement, with the assumption that *PDB-REDO* reflects the data-processing capabilities typical of an experimenter. A selection of 270 PDB entries, each with confirmed NEMOs in the deposited amplitudes, were chosen for this analysis (Supplementary Information S3, list of data sets and statistics). A comparison was conducted between the original deposited data sets and their respective counterparts with NEMOs removed. The deposited coordinates were randomized using *phenix.pdbtools* (Adams *et al.*, 2010 ▸), with a 0.25 Å shake applied. The default settings of *PDB-REDO* were consistently applied across all re-refinement processes.

## 3. Results and discussion

### 3.1. Performance of the NEMO-detection algorithm

Table 2 ▸ gives a comprehensive comparison of different NEMO-detection approaches. When Wilson statistics-based rejection (the *beamstop_shadow_outlier* function in *cctbx*) is employed the performance is less than satisfactory, as suggested by the four mediocre metrics in the first two rows. Two major factors contribute to this suboptimal performance: firstly, weak observations are not unusual for a diffraction data set even at low angle. The number of false positives hence becomes high with a loose probability threshold using Wilson statistics. Secondly, the normalization of amplitudes/intensities is susceptible to interference from absolute scaling, which is potentially affected by the binning strategies applied as well as the number of outliers within bins (Murshudov *et al.*, 1997 ▸; Read & McCoy, 2016 ▸). This consequently leads to an elevated rate of false negatives. The abundance of false positives pinpoints the risk of unnecessarily discarding valuable information, while an excess of false negatives poses challenges for the downstream structure solution. Merely adjusting the threshold for the cumulative probability (the parameter *t*) is ineffective to enhance evaluation, as all metrics remain relatively stagnant.

**Figure 4:**
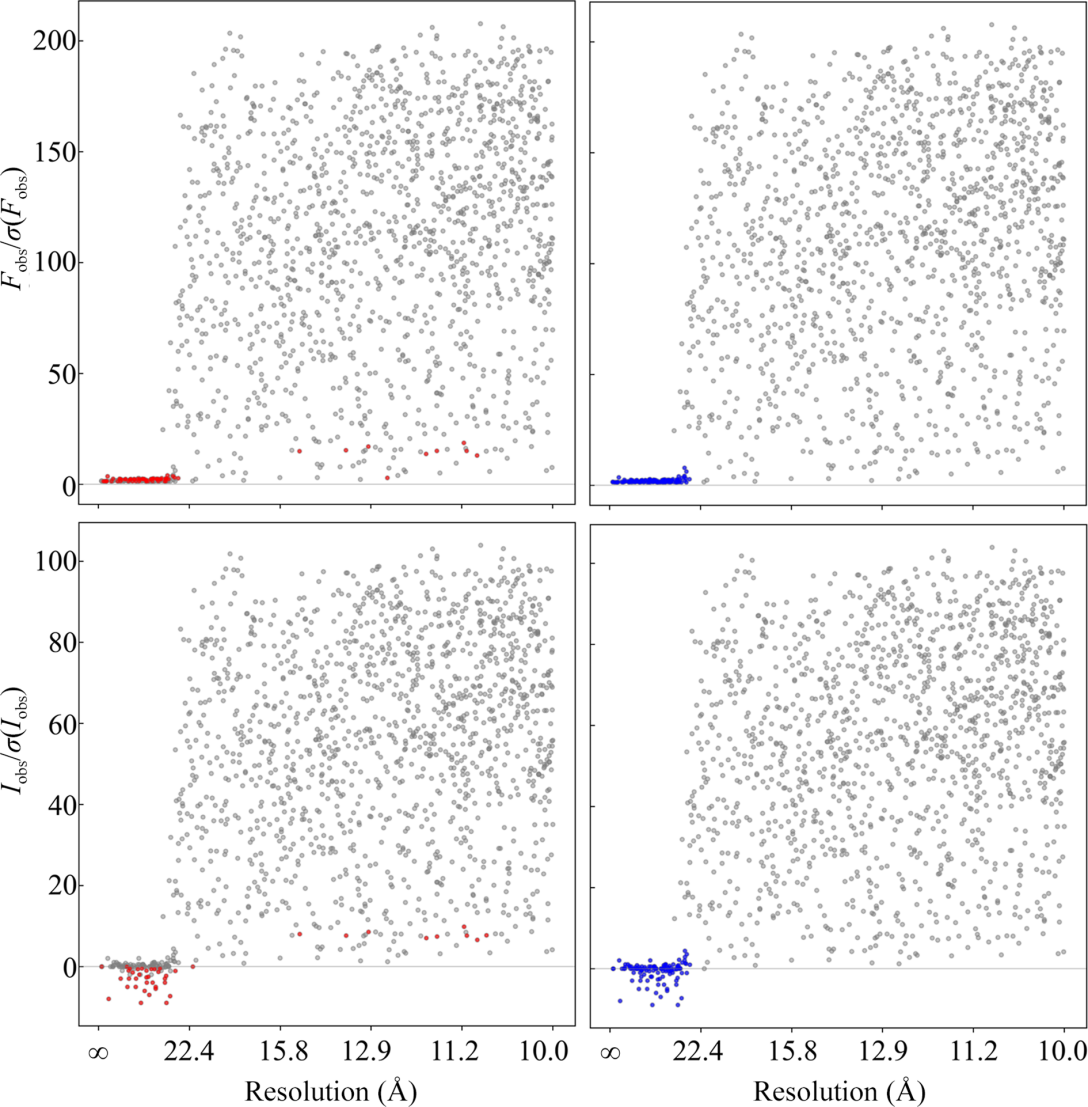
*AUSPEX* plots of the low-angle data subset of PDB entry 8g0s. Top: *F*_obs_/σ(*F*_obs_). Bottom: *I*_obs_/σ(*I*_obs_). Red dots are beamstop shadow outliers identified by statistical tests (equations 2–5) alone with a threshold of 10^−2^. Blue dots are NEMOs identified by our method derived from semi-supervised clustering. Observations that are not NEMOs can be falsely identified as outliers by statistical tests. Observations that are NEMOs can escape the statistical tests due to too many outliers disrupting the absolute scaling. Decreasing the threshold leads to an increased rate of false negatives. In this instance, our clustering-derived method successfully identified all NEMOs with perfect metrics.

Our clustering-derived method, integrating an additional and independent logical layer alongside statistical inference, demonstrates a relatively good performance (see Table 2 ▸ and the example in Fig. 4 ▸). Particularly for inputs free of twinning and tNCS, this method can reliably label NEMOs. When utilizing intensities, the performance is slightly worse compared with amplitude-based detection. We speculate that this difference may arise from the complexity of patterns in intensities compared with converted amplitudes, as hinted by the relatively lower specificity observed with intensity-based detection. The example in Fig. 4 ▸ shows that the ‘NEMO clusters’ in *I*_obs_/σ(*I*_obs_) are more dispersed than those in *F*_obs_/σ(*F*_obs_). This illustrates that the inferred amplitudes of weak signals could predominantly be influenced by the French–Wilson prior, potentially leading to the inadvertent loss of additional information implicitly encoded in intensities, including the sources of systematic errors.

It is important to note that the specificity of automatic detection can significantly diminish when twinning or tNCS are present. Since tNCS data sets inherently contain weak data with high errors (Read *et al.*, 2013 ▸), a substantial portion of the data are falsely flagged as outliers during the statistical testing phase, making the subsequent exclusion harder (Supplementary Fig. S3). In a twinned data set, intensity dispersion is lower compared with a generic data set (Stanley, 1972 ▸). Consequently, an excessive number of irrelevant sub­clusters may be assigned during the noise-level bootstrapping, potentially increasing the number of false positives. This serves as yet another example of how strong patterns in atomic crystal structures such as tNCS and crystal twinning result in altered intensity statistics, which in turn will confuse methods that are not designed to handle them. Nevertheless, despite reduced specificity in identifying exact reflections as NEMOs in the presence of twinning or tNCS, the automatic detection can still accurately determine whether the data set contains any NEMOs, even under these conditions.

### 3.2. Impact of NEMOs on refinement

The selected NEMO-containing data sets for analysis cover a broad spectrum of experimental characteristics, including various resolutions, *R*_free_ values, numbers of unique observations per non-H atom, solvent contents, synchrotron beamlines and data-processing software used (Table 3 ▸). The diverse synchrotron sources and data-reduction techniques imply that the presence of NEMOs is not uncommon, despite the robust implementation of explicit outlier-rejection methods in the current data-processing pipelines.

**Figure 5:**
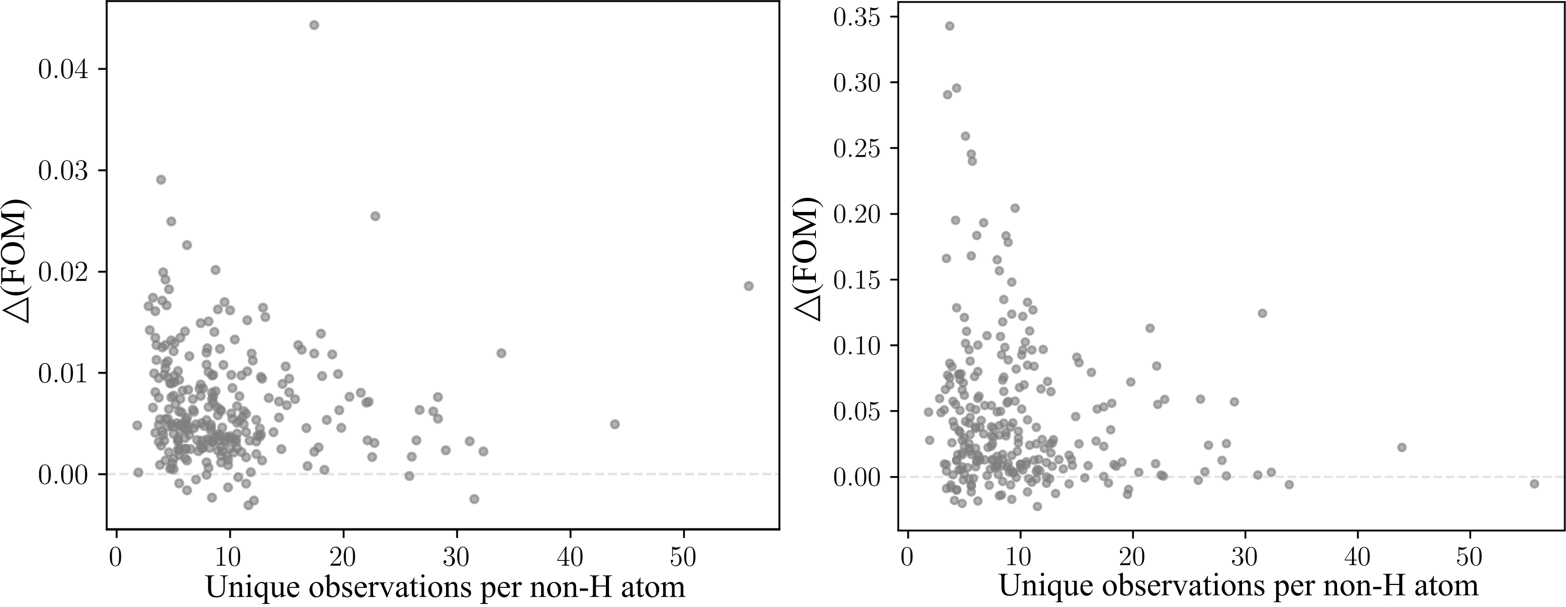
The difference in FOM [Δ(FOM)] at the end of re-refinement after and before NEMO removal for the selected data sets. Left: Δ(FOM) of the entire data set. Right: Δ(FOM) of the low-resolution shell. Note that the scales of the two panels are different. We consider that the number of unique observations per non-H atom remains approximately consistent before and after NEMO removal, given the small proportion of NEMOs (a maximum of 0.75% of the entire data set).

It is widely believed that once the structure has successfully been phased, low-angle observations have a minimal influence on model quality. The differences in figure of merit (FOM; Read, 1999 ▸; Murshudov *et al.*, 1997 ▸) across the entire data set and within the low-resolution shell at the end of refinement, after and before NEMO removal, suggest otherwise (Fig. 5 ▸). Despite the relatively small absolute fraction of NEMOs, the result clearly indicates that removing NEMOs from the data set effectively enhances phase quality, benefiting not only the low-resolution bin but also the entire data set. Especially in instances where the data-to-parameter ratio is low (*i.e.* fewer unique observations per non-H atom), attempting refinement with a data set containing NEMOs is more likely to impede the refinement process. In one illustrative example, we observed that ignoring the presence of NEMOs caused the molecular envelope to be flipped over, which shows systematically underestimated electron density within the protein envelope and overestimation of the bulk-solvent region density (Supplementary Fig. S6). Such distortion may affect the accuracy of the model built using such electron density, particularly the ligands at the protein–solvent interface.

### 3.3. Limitations and further implementation

The clustering-derived automatic detection of NEMOs has certain limitations. The performance deficiency in handling integrated intensities should be addressed, as intensities are more primordial than amplitudes. One potential solution is to use a more stable estimation of expected intensities, such as that implemented in *Phaser* (McCoy *et al.*, 2007 ▸). Another solution is to incorporate more intensity data into the training set, possibly by re-integrating diffraction images without the beamstop mask using various data-reduction software. With regard to data sets containing twinning or tNCS, improved sets for ***O*** and consequently better NEMO assignment may be achieved by correcting for the statistical effect of twinning and tNCS (for example using the tNCS-corrected ɛ factor; McCoy *et al.*, 2005 ▸). In addition, the extent to which this method is affected by other coexisting pathologies, such as improper Lorentz correction, remains unexplored. It is important to note that the selection of data sets for hyperparameter tuning and performance testing was not entirely random. This is because for some initially selected data sets it was not possible to reconstruct the ground-truth set due to issues with indexing during re-integration caused primarily by discrepancies in reported space group or unit-cell parameters. Nevertheless, we believe that this method, together with the idea of utilizing the strength of both crystallographic statistics and machine learning, will be widely useful for structural biologists and at synchrotron beamlines for macromolecular crystallography.

The automatic NEMO-detection feature has been incorporated into *AUSPEX* as well as the web service https://auspex.de and integrated into the *AUSPEX* plots presented to the user (Supplementary Figs. S4 and S5). Additionally, if necessary, the *AUSPEX* command-line interface can remove rows corresponding to NEMOs from an input reflection file before structure solution. In the current version, *AUSPEX* can be used to generate a list of reflections that should be ignored during scaling if unmerged reflection files are also provided. For example, when provided with an INTEGRATE.HKL file from *XDS*, *AUSPEX* will attempt to generate a FILTER.HKL file to prevent the CORRECT step of *XDS* from erroneously rejecting correctly recorded observations due to the presence of NEMOs within a group of symmetry-equivalent observations.

The tuned NEMO-detection algorithm has been implemented as the *NEMO* module within *AUSPEX* (https://github.com/thorn-lab/AUSPEX) and is available under the GNU Lesser General Public Licence, adhering to FAIR principles (Wilkinson *et al.*, 2016 ▸). The code efficiently handles noise-level bootstrapping in parallel. Within the *CCP*4 (version 8.0.019) virtual environment (Agirre *et al.*, 2023 ▸), most calculations are completed in less than a second on a 3 GHz CPU. The primary runtime bottleneck is the construction of tree structures for the calculation of ***C**_i_*_,*j*_, which accounts for 77% of the total runtime. There are no known memory bottlenecks.

## 4. Conclusion

The emergence of NEMOs in the merged data set mainly results from unmasked/partially masked beamstops and the scaling protocols employed during data reduction. While detecting a few weak outliers using global data-quality indicators is challenging, the pattern posed by systematic errors, such as NEMOs, is discernible through direct observation of the data in a certain form (for example an *AUSPEX* plot). By combining statistical inference with machine-learning concepts such as clustering and hyperparameter tuning, we have developed an explainable model to identify and exclude NEMOs with better reliability than was previously possible. Our approach suggests that by accurately pinpointing the source and recognizing the pattern of the corresponding error (which is only possible with a sufficient amount of raw data deposition), it becomes feasible to exclude such errors during automatic data processing.

As the recent novel Bragg peak-finding algorithm based on machine learning does not completely exclude NEMOs (Dong *et al.*, 2024 ▸), we would like to emphasize that the optimal practice to eliminate NEMOs is to mask the untrusted region properly before data reduction. However, achieving zero NEMOs while minimizing information loss with an optimally modelled beamstop mask is a sophisticated task. As far as we are aware, there is no universally accepted convention for generating an objectively perfect mask for the whole data set (Lyubimov *et al.*, 2016 ▸), as this process often involves trial and error (for example iteratively adjusting the lower bound of trusted detector pixels in *XDS* and inspecting the respective background table). Moreover, data derived from manually masked images do not consistently enhance the fit between model and data due to the loss of low-resolution information (Supplementary Table S1 and Supplementary Information S4), and until now there has been no reliable method to detect NEMOs in processed data sets. Our method serves as a promising tool to evaluate the goodness of a beamstop mask in retaining as much low-resolution information as possible without interrupting any existing data-reduction pipeline. Specifically, the number of NEMOs at the end of the data-reduction process can be used as an objective indicator to iteratively model a beamstop mask that is sufficient yet not overly extensive for the entire data set. This approach is superior to manually generated polygons, which typically rely on only one or a few reference frames, and to the provided metadata, which may be inadequate for the whole data set as other sources that affect the assignment of shadowed pixels may remain unrecognized. In a time-resolved and serial crystallography setup where adapting a static beamstop mask is in general impractical, our method can be useful in the post-mortem exclusion of NEMOs in integrated data sets, given that the unmerged data are not often available. The approach proposed here also holds practical utility for adapting to the evolving detection strategies of next-generation X-ray diffraction experiments as well as beamline automation.

## 5. Related literature

The following references are cited in the supporting information for this article: Bergstra *et al.* (2011 ▸), Emsley *et al.* (2010 ▸) and Hubert & Arabie (1985 ▸).

## Supplementary Material

Supplementary Information S1.1. DOI: 10.1107/S2059798324008519/rr5246sup1.txt

Supplementary Information S1.2. DOI: 10.1107/S2059798324008519/rr5246sup2.txt

Supplementary Information S2.1. DOI: 10.1107/S2059798324008519/rr5246sup3.txt

Supplementary Information S2.2. DOI: 10.1107/S2059798324008519/rr5246sup4.txt

Supplementary Information S3. DOI: 10.1107/S2059798324008519/rr5246sup5.txt

Log files for refinement and re-integration. DOI: 10.1107/S2059798324008519/rr5246sup6.zip

Supplementary Figures and Tables, inlcuding annotation for Supplementary Information files. DOI: 10.1107/S2059798324008519/rr5246sup7.pdf

## Figures and Tables

**Table 1:** Definitions of true positive (TP), false positive (FP), true negative (TN) and false negative (FN)

	∈ ***N***	∈ ***G***	∈ ***O***′†
TP	True	True	—
FP	True	False	—
TN	False	False	True
FN	False	True	—

† ***O***′ := {(*x*, *y*)|*x* < 0.01 Å^−2^ and *p*[*E*(*y*)] < 0.03}.

**Table 2:** Comparison of different beamstop shadow outlier-detection algorithms

	Input	Precision	Accuracy	Sensitivity	Specificity
*beamstop_shadow_outlier* in *cctbx*	*F*_obs_	0.787 ± 0.045	0.692 ± 0.051	0.750 ± 0.048	0.569 ± 0.055
	*F*_obs_ †	0.809 ± 0.047	0.729 ± 0.053	0.794 ± 0.048	0.584 ± 0.058
Clustering-derived detection	*F*_obs_/σ(*F*_obs_)	0.927 ± 0.029	0.912 ± 0.031	0.928 ± 0.029	0.887 ± 0.035
	*F*_obs_/σ(*F*_obs_)†	0.974 ± 0.019	0.960 ± 0.023	0.959 ± 0.023	0.960 ± 0.023
Clustering-derived detection	*I*_obs_/σ(*I*_obs_)	0.935 ± 0.046	0.901 ± 0.056	0.932 ± 0.047	0.814 ± 0.072
	*I*_obs_/σ(*I*_obs_)†	0.958 ± 0.040	0.935 ± 0.050	0.955 ± 0.042	0.875 ± 0.067

† The test was made excluding data sets with twinning or tNCS.

**Table 3:** The characteristics of the 270 data sets selected for re-refinement (details in Supplementary Information S3)

Experimental characteristics	Range
Resolution (Å)	3.38–0.97
*R*_free_	0.341–0.119
No. of unique observations per non-H atom	1.8–55.7
Solvent content (%)	32.25–81.83
Synchrotron sources	NSLS-II, ESRF, BESSY, PETRA III, APS, Diamond, SSRL, Australian Synchrotron, ALS, LNLS
Data-reduction tools	*autoPROC* (Vonrhein *et al.*, 2011 ▸), *DENZO* (Otwinowski & Minor, 1997 ▸), *DIALS* (Winter *et al.*, 2018 ▸), *HKL*-2000, *HKL*-3000 (Minor *et al.*, 2006 ▸), *MOSFLM* (Battye *et al.*, 2011 ▸), *XDS* (Kabsch, 2010 ▸)
NEMOs (%)	0.75–0.0015
