# Supplementary material for: Robust and automatic beamstop shadow outlier rejection: combining crystallographic statistics with modern clustering under a semi-supervised learning strategy
Source: Acta Crystallogr D Struct Biol. 2024 Oct 1;80(Pt 10):722–32. doi: 10.1107/S2059798324008519 (PMC11448920; doi:10.1107/S2059798324008519)
Supplement: Supplementary file 7 [file d-80-00722-sup7.pdf]

STRUCTURAL BIOLOGY

Volume 80 (2024)

Supporting information for article:

Robust and automatic beamstop shadow outlier rejection: combining crystallographic statistics with modern clustering under a semi-supervised learning strategy

Yunyun Gao, Helen M. Ginn and Andrea Thorn

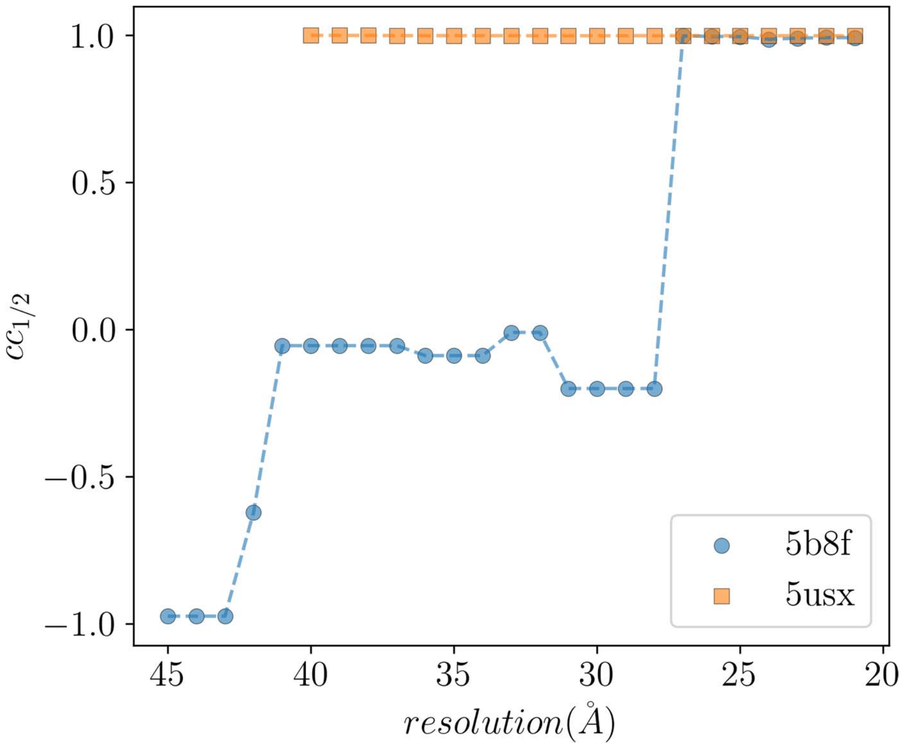

Figure S1 $CC_{1/2}$ of two re-integrated datasets containing beamstop shadow outliers. $CC_{1/2}$ is computed with the method outlined in (Assmann *et al.*, 2016). Each point in the figure is calculated by incorporating all observations below its corresponding resolution. In the case of PDB entry 5usx, no low-angle data should be omitted according to $CC_{1/2}$. For PDB entry 5b8f, setting a low-resolution cutoff at 27.5 Å results in the exclusion of 9 beamstop shadow outliers and 12 well-behaved unique observations from the merged dataset, causing unwanted loss of information.

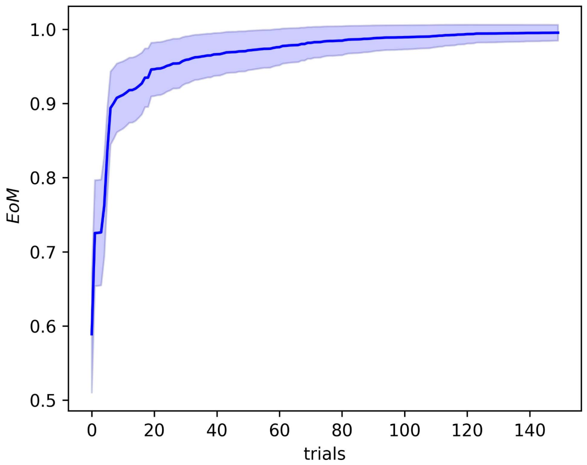

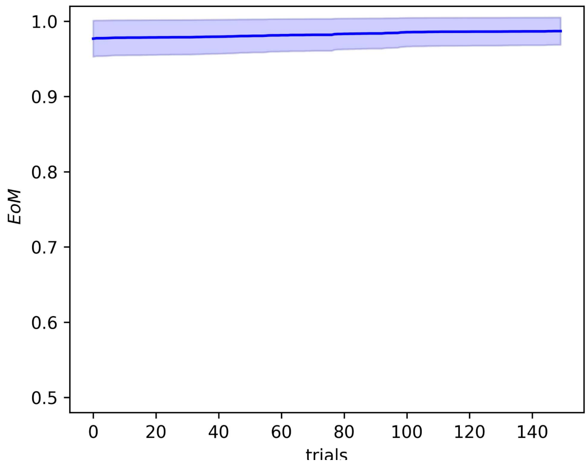

Figure S2 Hyperparameter optimization via expectation of maximum performance (*EoM*). Let ϕ be a set of hyper parameters, $\mathcal{P}$ the performance of the algorithm given ϕ, and $\mathcal{M}(\ldots)$ an evaluation metric for the assessment of $\mathcal{P}$. $EoM = \max \mathcal{M}(\mathcal{P}(\boldsymbol{A}, \phi), \boldsymbol{G})$, where $\boldsymbol{A}$ represents the datasets and $\boldsymbol{G}$ denotes the ground truth. The metric $\mathcal{M}(\ldots)$ refers to the modified Rand index (Hubert & Arabie, 1985). To optimize *EoM*, we employed Monte Carlo sampling in the simplest case for the input set, $F_{obs}/\sigma(F_{obs})$ versus inverse *d*-spacing squared (corresponding labels are provided in Supplementary Information S1.1). At the end of the trials, ϕ_{amp} was determined. Afterwards, TPE (Tree-structured Parzen Estimator) sampling (Bergstra *et al.*, 2011) using ϕ_{amp} as the starting point was conducted for the input set, $I_{obs}/\sigma(I_{obs})$ versus inverse *d*-spacing squared (corresponding labels are Supplementary Information S1.2). ϕ_{int} was determined at the end of the following trials. Figures on the left and right show the evolution of *EoM* in determining ϕ_{amp} and ϕ_{int}, respectively. The 95% confidence intervals are depicted as the filled area.

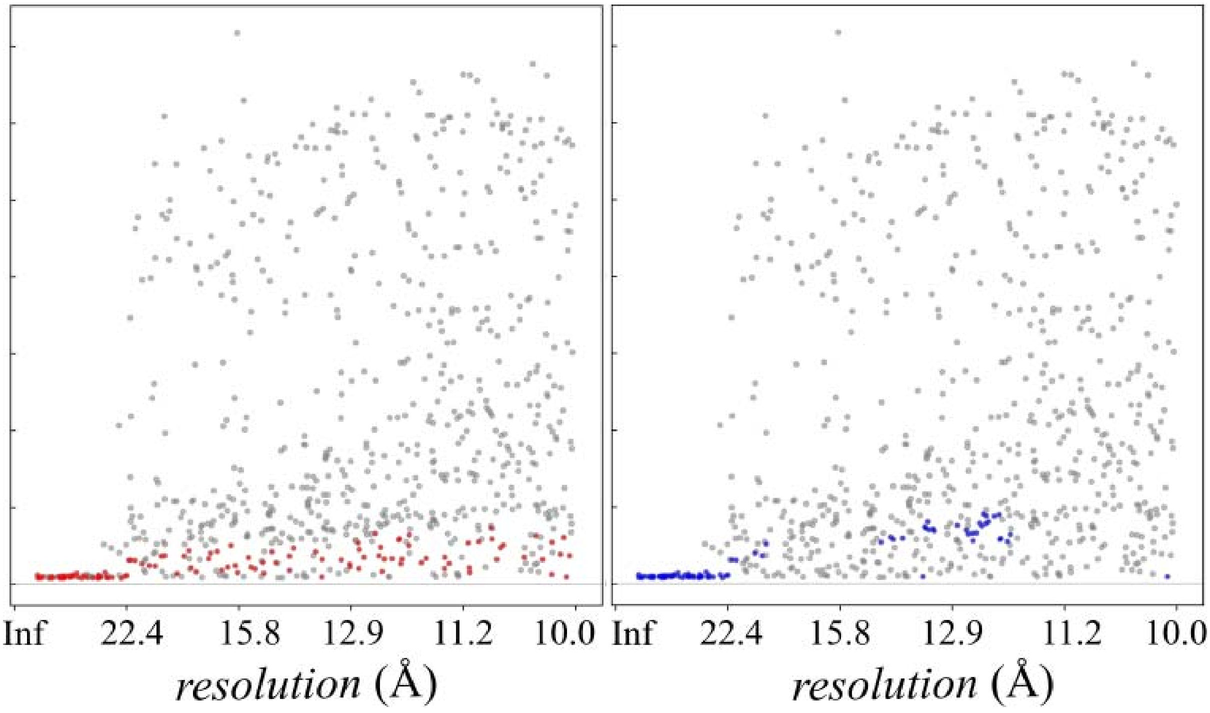

Figure S3 The AUSPEX plot of the low-angle data subset of 8ek7. The dataset has been tested (*Xtriage*) to exhibit pseudo translational symmetry. In this particular case, a substantial portion of the reflections, that are not Not-Excluded-unMasked-Outliers (NEMOs), are flagged as outliers during the statistical testing phase. A portion of the same density to the flagged data is falsely categorized as NEMOs during the subsequent clustering phase.

Table S1 Comparison of data features and structure solution between different protocols

	protocol	lowest resolution (Å)	low-res shell completeness	Rwork(Rfree)
5b8f	default mask+NEMO removal	50.2	0.75	0.1397 (0.1674)
	complete mask	37.3	0.50	0.1403 (0.1682)
5usx	default mask+NEMO removal	45.5	0.95	0.1846 (0.2249)
	complete mask	43.9	0.85	0.1912 (0.2287)

The default mask+NEMO removal protocol is conducted as follows: (1) Data reduction with XDS using the default DEFPIX settings. (2) Convert reflection data files to MTZ using XDSCONV. (3) Remove NEMOs from the MTZ. (4) Conduct refinement using PDB-REDO.

The complete mask protocol is conducted as follows: (1) Data reduction with XDS using a manually generated beamstop mask using the dispersion plot in the *dials.image_viewer* as reference. (2) Convert reflection data files to MTZ using XDSCONV. (3) Conduct refinement using PDB-REDO.

Re-integration was conducted in the same way as described in 2.2. The highest resolution cutoff is set the same as the deposited value. Refinement was conducted using PDB-REDO in the same way as described in 2.3. The corresponding XDS.INP and PDB-REDO logs are deposited as a part of the supplementary information (Supplementary Information S4).

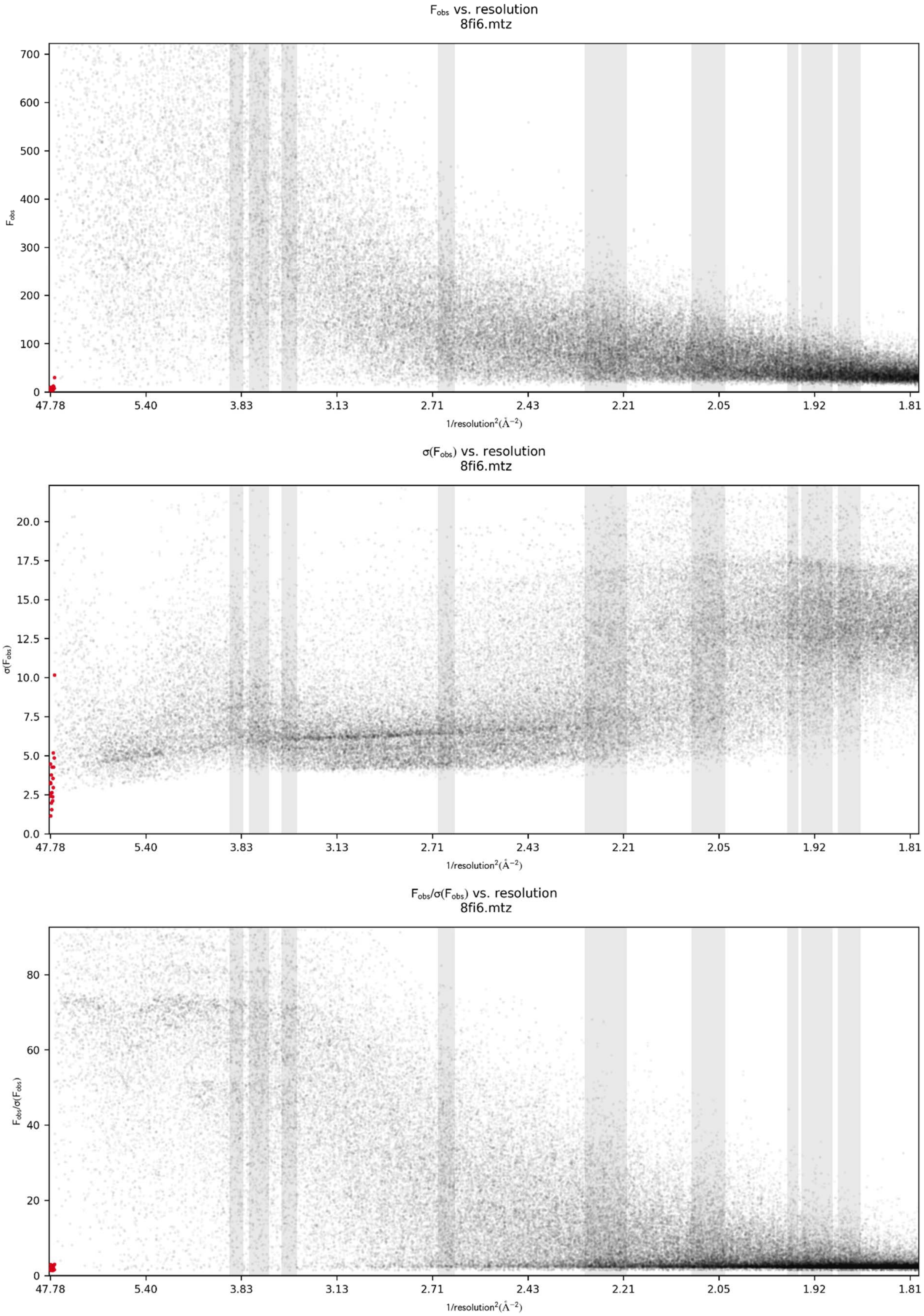

Figure S4 Example of AUSPEX amplitude plots with --*beamstop-outlier* flag on.

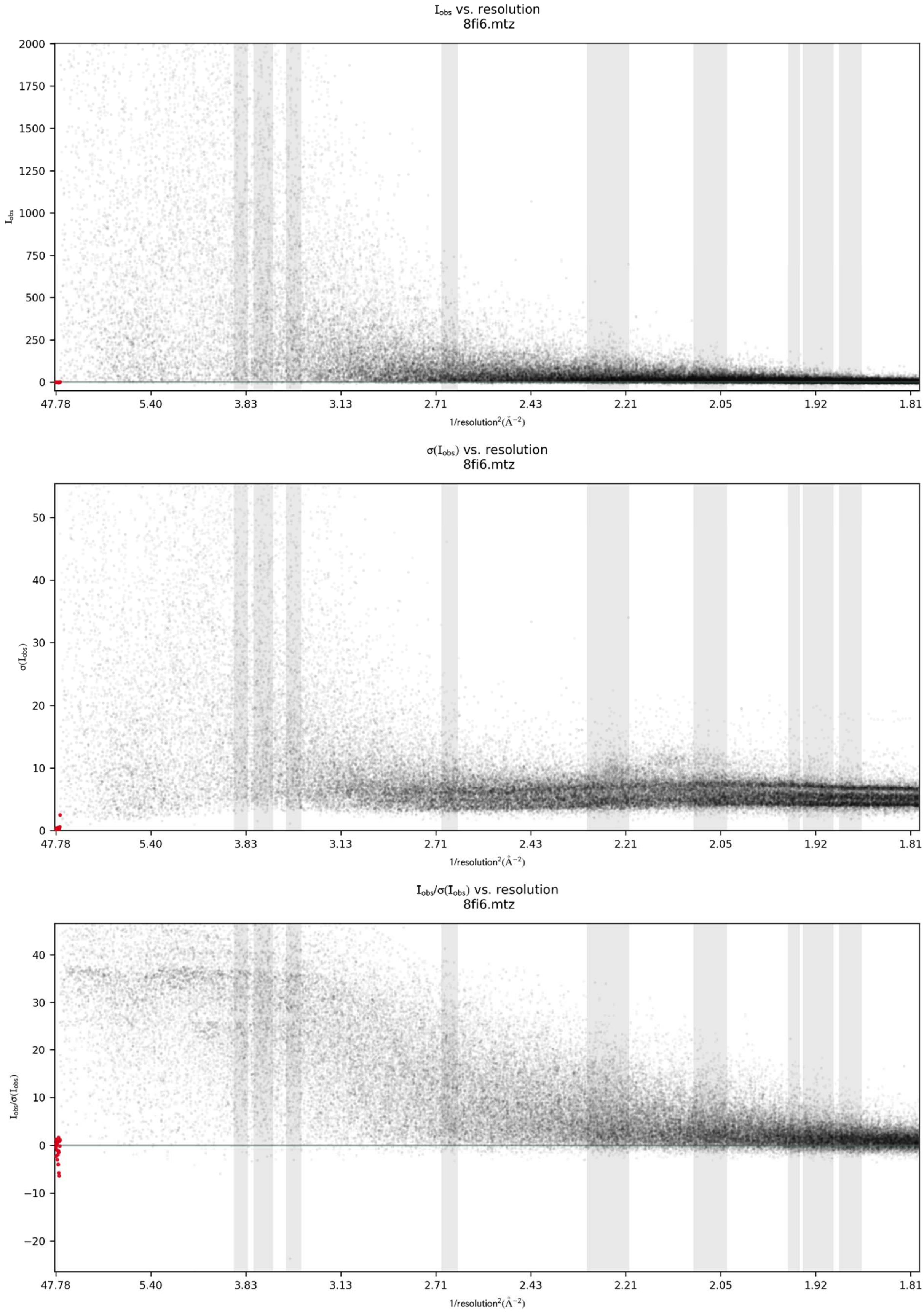

Figure S5 Example of AUSPEX intensity plots with --*beamstop-outlier* flag on.

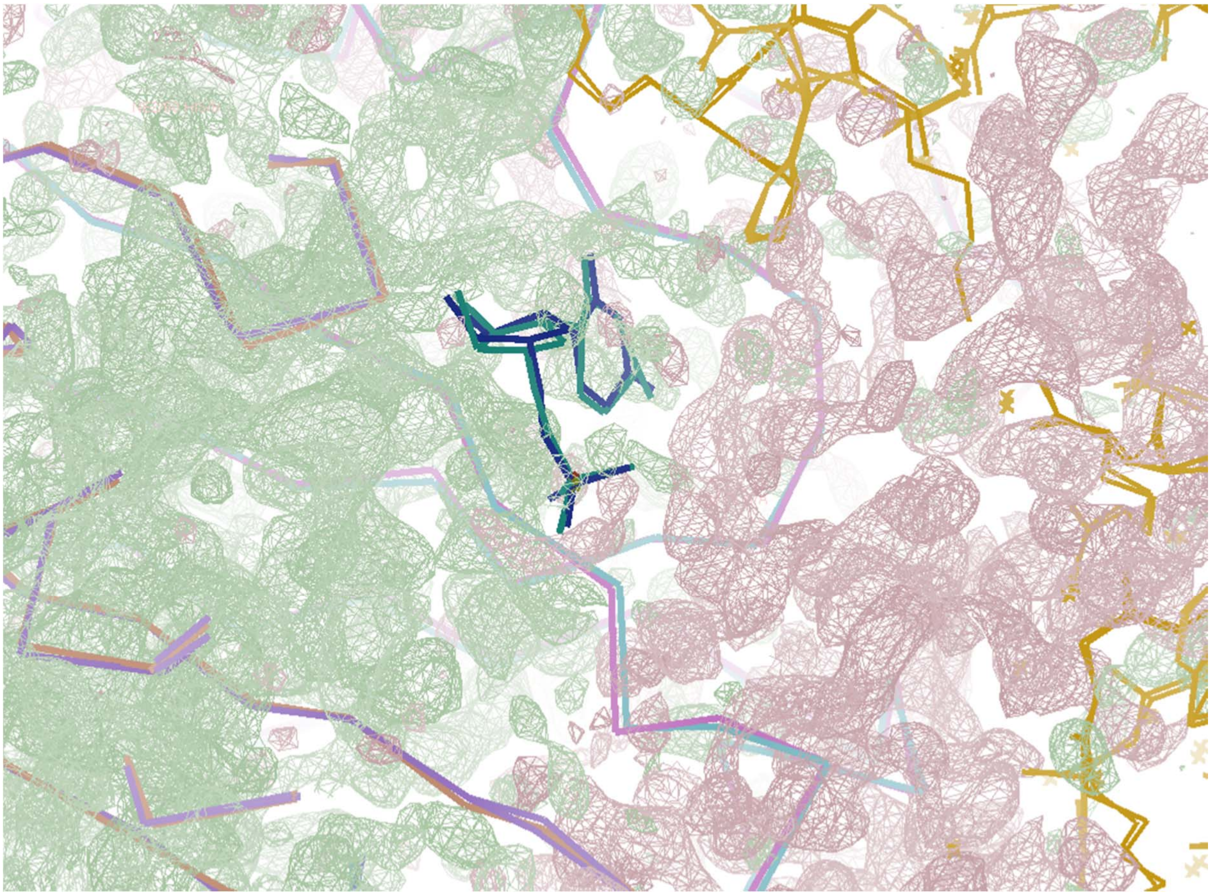

Figure S6 A comparison between PDB-redo re-refinement results of 5b8f using the NEMO-containing dataset and the NEMO-excluded dataset. The difference map (NEMO-containing − NEMO-excluded) is contoured at 0.03 e/Å^{-3} using Coot (Emsley *et al*., 2010). Refinement using the dataset with NEMO artifact tend to systematically underestimate the electron density within the protein envelope and overestimate on that of the bulk solvent region. This may affect the model-building of the ligand at the solvent interface (blue/cyan backbones) and potentially the biological interpretation of it.

Table S2 Annotation for the Supplementary Information files

	Description	Column 1	Column 2	Column 3	Column 4
S1.1	the ground truth set for hyperparameter tuning, amplitude version	PDB ID	Labels for all valid low-angle unique reflections (*d*-spacing > 10 Å) sorted by *d*-spacing (high to low). Label 0: the corresponding reflection is not a beamstop outlier. Label 1: corresponding reflection is assigned as a beamstop outlier.		
S1.2	the ground truth set for hyperparameter tuning, intensity version	PDB ID	Labels for all valid low-angle unique reflections (*d*-spacing > 10 Å) sorted by *d*-spacing (high to low). Label 0: the corresponding reflection is not a beamstop outlier. Label 1: corresponding reflection is assigned as a beamstop outlier.		
S2.1	the ground truth set for performance test, amplitude version	PDB ID	The row indices of beamstop outliers identified in the deposited data. Empty list means no beamstop outliers.	twinning?	tNCS?
S2.1	the ground truth set for performance test, intensity version	PDB ID	The row indices of beamstop outliers identified in the deposited data. Empty list means no beamstop outliers.	twinning?	tNCS?

S3 contains detailed characteristics of the 270 datasets selected for re-refinement. The column labels are given in the column headers.

References

Assmann, G., Brehm, W. & Diederichs, K. (2016). *J. Appl. Crystallogr.* **49**, 1021–1028.

Bergstra, J., Bardenet, R., Bengio, Y. & Kégl, B. (2011). *Advances in Neural Information Processing Systems*.

Emsley, P., Lohkamp, B., Scott, W. G. & Cowtan, K. (2010). *Acta Crystallogr. D Biol. Crystallogr.* 66, 486–501.

Hubert, L. & Arabie, P. (1985). *J. of Classification*. **2**, 193–218.
